# Intraoperative Thrombophilia-Associated Thrombosis of Both Saphenous Veins during Harvesting for Coronary Artery Bypass Grafting

**DOI:** 10.1055/s-0040-1715657

**Published:** 2020-08-23

**Authors:** Piotr Mazur, Michał Ząbczyk, Radosław Litwinowicz, Joanna Natorska, Bogusław Kapelak

**Affiliations:** 1 Institute of Cardiology, Jagiellonian University Medical College, Krakow, Poland; 2 Department of Cardiovascular Surgery and Transplantology, The John Paul II Hospital, Krakow, Poland

**Keywords:** coronary artery bypass grafting, thrombosis, fibrin, fibrinogen, mutation

## Abstract

**Introduction** Intraoperative thrombosis of saphenous veins (SV) during open harvesting is very rare.

**Case Report** We present a case of a 60-year-old male patient with multivessel coronary artery disease and a history of a non-ST elevation acute coronary syndrome, and type-2 diabetes mellitus admitted for coronary artery bypass grafting, in whom bilateral intraoperative SV thrombosis occurred during graft harvesting. Routine thrombophilia screening showed no abnormalities and cancer was excluded. Compared with healthy controls, we observed prolonged fibrin clot lysis time and increased thrombin generation reflected by endogenous thrombin potential. Scanning electron microscopy of the thrombosed material revealed compact and thick fibrin layer on the clot surface with a solid mass of unusually compressed platelets and erythrocytes underneath. The patient was tested for fibrinogen and factor (F) XIII polymorphisms, and was found to be heterozygous for β-fibrinogen HaeIII (-455G > A) and FXIII Val34Leu (100G > T).

**Conclusion** β-fibrinogen HaeIII and FXIII Val34Leu polymorphisms are reflected in reduced clot permeability and susceptibility to lysis, and might contribute to intraoperative SV thrombosis during vascular grafting procedures. Carriers of those are at risk of primary venous graft failure after bypass procedures.

## Introduction

Coronary artery bypass grafting (CABG) is a method of choice for revascularization in patients with multivessel disease and diabetes mellitus (DM). Although arterial grafts are preferred in selected scenarios, the common practice is to use left internal thoracic artery (LITA) to bypass the left anterior descending artery (LAD), and to place venous conduits to other target vessels. An often chosen vascular graft, the great saphenous vein (SV), offers decent durability and is easy to harvest. SV graft occlusion may occur in up to 12% of cases within the first 6 months, and as many as 3.4% may occlude within first 2 to 3 weeks. 1 SV harvesting dramatically changes the vein's environment with disruption of blood flow in vasa vasorum, damage to the adventitia, hypoxia, and hyponutrition of the vessel wall along with focal endothelial disruption. 2 Acute SV graft failure is usually a result of graft thrombosis which, among other factors, like technical failure, graft-target vessel disproportion etc., may be caused by hypercoagulability.

## Case Report

A 60-year-old male patient with multivessel coronary artery disease, who suffered from a non-ST elevation acute coronary syndrome (NSTE-ACS) 1 month prior to admission, a nonsmoker with type-2 DM on metformin, peptic ulcer disease, and a history of alcohol abuse, was admitted to our institution for CABG. Just after the NSTE-ACS, a left ventricle (LV) thrombus was seen on one echocardiographic examination, but it was absent during follow-up. There was no deep venous thrombosis or bleeding diathesis history. On admission, the patient was on aspirin 75 mg (once daily) and enoxaparin 60 mg (once daily). Routine laboratory tests were within normal ranges ( Table 1 ). There were no abnormalities on physical examination, apart from obesity (body mass index, 32.7 kg/m ^2^ ) when the patient was admitted. The lower extremities appeared normal. There were no varicose veins, no signs or symptoms of venous insufficiency, and the past medical history was negative for both personal and family history of chronic venous insufficiency or varicose veins. The patient was operated on following the standard procedures. During LITA harvest, a cardiac surgery resident harvested the right SV using the open technique. The wall of the SV looked grossly normal. Upon dissection, the side branches were tied off and clipped, and a needle was placed at the distal end, while the proximal end was still not separated. An attempt was made to flush the vein with a solution containing blood (20 mL), heparin (15,000 IU), and normal saline (10 mL), while the distal end was closed with an atraumatic vascular clamp, and vein thrombosis was noted. Upon the separation of the distal end, a luminal thrombus was visible. The left SV was then taken down using the same protocol by an experienced staff cardiac surgeon, with the same result. Presence of a luminal thrombus was confirmed upon separation of the proximal end. Systemic heparin was administered, and normal LITA outflow was confirmed. Concerns regarding safety of cardiopulmonary bypass use were raised due to suspected thrombotic issue, and the approach was modified. The LITA–LAD anastomosis was completed off-pump on a beating heart.

**Table 1:** Results of initial and follow-up laboratory testing

Variable	Normal ranges	Preoperative	Postoperative day 6	Postoperative day 103
Coagulation tests				
Red blood count (10 ^3^ /µL)	4.20–6.00	4.00	3.36	–
Hemoglobin (g/dL)	14.0–18.0	9.7	8.5	–
White blood count (10 ^3^ /µL)	3.80–10.00	7.19	6.55	–
Platelet count (10 ^3^ /µL)	140–440	296	287	–
APTT, s	25.9–36.6	30.2	28.2	–
PT (s)	10.4–13.0	12.3	11.4	–
PT (%)	82–121	86	99	–
INR	<1.50	1.11	1.02	–
Platelet aggregation				
0.5 mmol/L arachidonic acid, %	–	–	54	–
10 µmol/L ADP, %	–	–	52	–
Thrombophilia screening				
Fibrinogen (g/L)	2.10–4.00	–	6.32	4.10
Antithrombin III (%)	79–112	–	89	89
D-dimer (µg/L)	<500	–	1825	–
anti-Xa (IU/mL)	–	–	0.48	–
Homocysteine (µmol/L)	3–15	–	11.5	12.4
Protein C (%)	70–140	–	123	119
Protein S (%)	67–139	–	70	70
Factor VIII (%)	70–150	–	291	135
Leiden c.1601G > A	–	–	GG (no mutation)	GG (no mutation)
Prothrombin c.*97G > A	–	–	GG (no mutation)	GG (no mutation)
β-fibrinogen -455G > A	–	–	–	GA (heterozygote)
Factor XIII 100G > T	–	–	–	GT (heterozygote)
Lupus anticoagulant ratio	<1.20	–	1.06	0.86
Lupus anticoagulant ratio (APTT)	<1.15	–	0.93	0.85
Anticardiolipin IgG	0–15	–	3.4 GPL	5.4 GPL
Anticardiolipin IgM	0–17	–	3.5 MPL	2.7 MPL
Anti-β-2-glycoprotein I IgG antibody	0.0–8.0	–	1.0 SGU	0.5 SGU
Anti-β-2-glycoprotein I IgM antibody	0.0–10.0	−	1.2 SMU	1.0 SMU

Abbreviations: APTT, activated partial thromboplastin time; GPL, IgG phospholipid unit; Ig, immunoglobulin; INR, international normalized ratio; MPL, IgM phospholipid unit; PT, prothrombin time; SGU, standard IgG β-2 glycoprotein unit; SMU, standard IgM β-2 glycoprotein unit.

The postoperative course was uneventful. On postoperative day 1, the patient received dual antiplatelet therapy with aspirin and clopidogrel and was discharged on day 6 with no signs of thrombosis or myocardial ischemia. Elective angioplasty of nongrafted vessels was scheduled, and a complete thrombophilia screening was done ( Table 1 ). On the 3- and 12-month follow-up, the patient did well.

## Diagnostic Approach

Because a thrombophilia was suspected, screening was initiated showing no abnormalities ( Table 1 ).

Cancer was excluded as a cause of thrombosis. Positive antibodies against neutrophil cytoplasm antigens (pANCA and cANCA) were excluded as a cause of vasculitis. We then proceeded to analyze fibrin phenotype, using the previously described methodology. 3 4 Briefly, plasma fibrin clot permeability was determined in a hydrostatic pressure system. Tubes containing fibrin clots formed from adding 20 mmol/L calcium chloride and 1 U/mL human thrombin (Sigma) to citrated plasma, were connected through plastic tubing to a buffer reservoir (0.05 M Tris-HCl, 0.15 M NaCl, pH 7.5). The volume flowing through the gel was measured within 60 minutes. A permeation coefficient (Ks), reflecting pore size, was calculated from equation: *K*_s_ = *Q* × *L* *η* / *t* × *A* × Δ *p* , where *Q* is the flow rate in time *t* , *L* is the length of a fibrin gel, *η* is the viscosity of liquid, *A* is the cross section area, and Δ *p* is a differential pressure in dyne/cm ^2^ . Lower *K* s values indicated reduced permeability. Fibrinogen was determined using the Clauss method. Even though the follow-up fibrinogen level was normal, we identified strongly decreased fibrin clot permeability ( *K*_s_ = 3.31 ± 0.15 ×10 ^−9^ cm ^2^ ), compared with healthy controls from our previous report ( *n* = 30, *K*_s_ = 7.55 [7.00–7.96] 10 ^−9^ cm 23 ; samples collected during late follow-up appointment on postoperative day 103). Compared with healthy controls ( *n* = 30) we observed prolonged clot lysis time (CLT; 101 ± 6 vs. 135 ± 4.5 minutes) and increased thrombin generation reflected by endogenous thrombin potential (ETP) in the studied subject (ETP = 1,138 ± 134 vs. 1,463 ± 87 nM × min, respectively); measurement of the thrombin generation was done with calibrated automated thrombography (thrombinoscope BV, Maastricht, the Netherlands) according to the manufacturer's instructions in the 96-well plate fluorometer (Ascent Reader, Thermolabsystems OY, Helsinki, Finland), equipped with the 390/460 filter set, at a temperature of 37°C. Briefly, 80 microliters of platelet-poor plasma were diluted with 20 µL of the reagent containing 5 pmol/L recombinant tissue factor, 4 micromolar phosphatidylserine/phosphatidylcholine/phosphatidylethanolamine vesicle, and 20 µL of FluCa solution (Hepes, pH 7.35, 100 nmol/L CaCl _2_ , 60 mg/mL bovine albumin, and 2.5 mmol/L Z-Gly-Gly-Arg-7-amino-4-methylcoumarin). Each plasma sample was analyzed in duplicate. For analysis, the maximum concentration of thrombin generated was used. 3

**Fig. 1:**
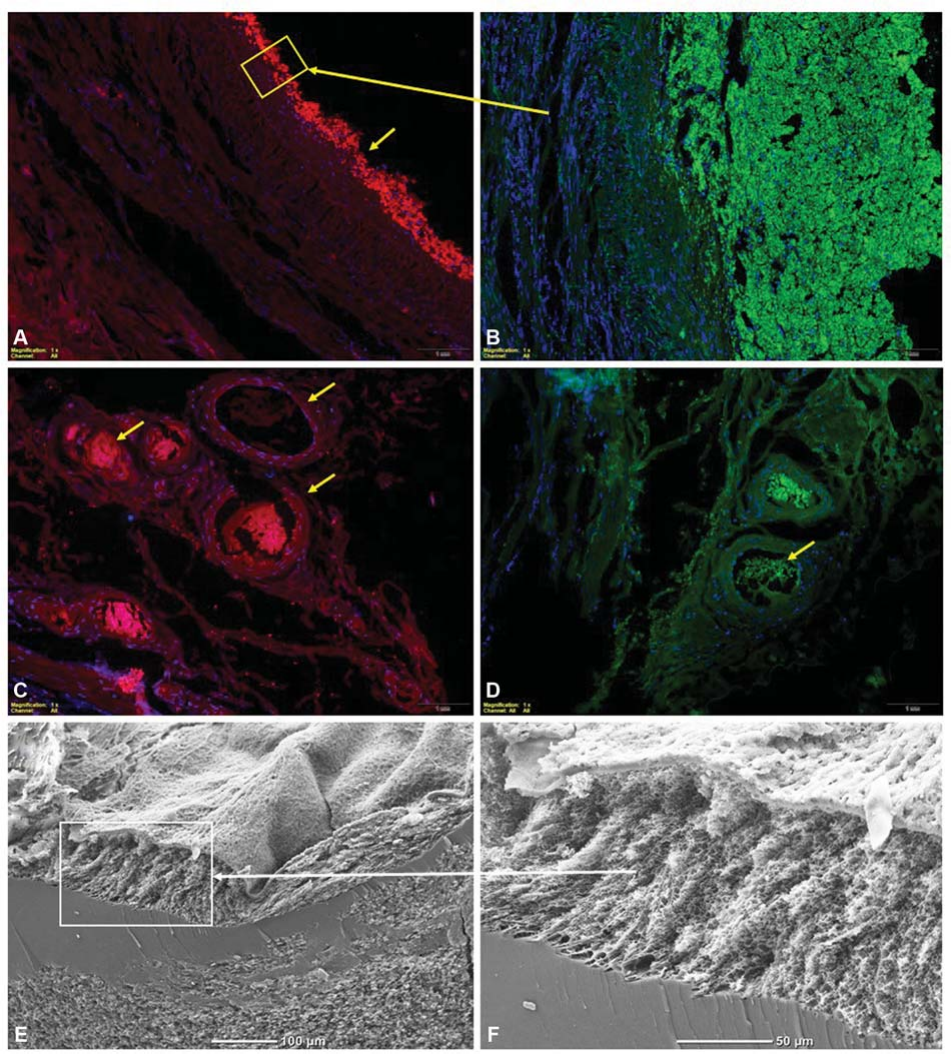
Representative images of SV graft immunostaining after massive thrombosis ( **A–D** ) prothrombin stained red, TF stained green, nuclei stained blue using DAPI and scanning electron microscopic images ( **E, F** ) of the surface of whole blood clot formed in vitro from citrated blood obtained from the patient undergoing CABG. Box and arrow represent magnification of the fragment in the box. Arrows show pertinent stained fragments (see text). CABG, Coronary artery bypass grafting; SV, saphenous veins; TF, tissue factor.

Cryosectioned tissue sections were fixed in ice-cold methanol–acetone (1:1) mixture, peroxidase activity was quenched with 3% H _2_ O _2_ and unspecific background was blocked with 3% bovine albumin (BSA, Sigma Co, St. Louis, Missouri, United States). Primary adequate antibodies against fibrin or tissue factor (TF) were applied (both Abcam, Cambridge, United Kingdom). Primary antibodies were followed by the corresponding secondary antibodies conjugated with fluorochrome (Abcam) as previously described. 5 Images were analyzed using Olympus BX 43 microscope. SVs immunostaining revealed thick layer of fibrin directly on the vessel endothelium ( Fig. 1A ) and high TF ( Fig. 1B ) activity. Within the thrombus we found abundant blood nuclear cells (nuclei stained using DAPI) suggesting the presence of proinflammatory monocytes, which are known source of TF. Unfortunately, we were not able to immunostain CD68 due to high unspecific background resulting from large amounts of fibrin. The microscopic analysis showed abundant adventitial vessels ( Fig. 1C, D ). Within almost every single vessel, we found thrombi rich in both prothrombin ( Fig. 1C ) and TF ( Fig. 1D ).

Prothrombotic fibrin clot phenotype reflected by reduced *K*_s_ and prolonged CLT along with enhanced thrombin generation and unusual images obtained from the immunostaining of the SVs prompted us to perform analysis of whole blood clot morphology using scanning electron microscopy (SEM), as previously described. 6 After washing, the thrombus was fixed with 2.5% glutaraldehyde phosphate buffered saline solution. Specimens were dehydrated, gold coated, and photographed digitally with a JEOL JSM 5410 (JEOL, Tokyo, Japan). The analysis revealed compact and thick fibrin layer on the clot surface ( Fig. 1 E , F ) with a solid mass of unusually compressed platelets and erythrocytes underneath. This observation suggested very-high contractile forces during clot formation in a platelet-driven, fibrin-mediated mechanism of clot contraction, and prompted us to study common fibrinogen and factor (F) XIII polymorphisms. The patient was heterozygous for β-fibrinogen HaeIII (-455G > A) and FXIII Val34Leu (100G > T).

## Discussion

A dramatic intraoperative SV thrombosis provoked by graft harvesting for CABG lead to change in revascularization strategy, but its cause remained unknown following the standard thrombophilia screening. The cases of acute SV graft thrombosis in the perioperative period are very rare, and as few as 3% of grafts occlude within first 2 to 3 weeks. 1 7

A normal SV is composed of the intima, the media, and the adventitia. 8 The intima is built of the layer of endothelial cells on the luminal side, the media consists of smooth muscle cells, and the adventitia forms the outer part. 8 In a normal setting, the endothelium is crucial for vein integrity and prevention of thrombosis, 9 and its focal disruption may predispose to vessel thrombosis. 2 SV manipulation and implantation leads to loss of endothelial integrity and elicits an inflammatory response with platelet adhesion and leukocyte recruitment. Notwithstanding, an overt thrombosis is extremely rare in the operating room. SV dissection results in blood flow disruption in vasa vasorum, and causes adventitial damage, hypoxia, and vessel wall hyponutrition. 10 Acute perioperative saphenous vein graft failure is almost always a result of graft thrombosis, but this very uncommonly occurs prior to graft placement. Surgical factors, like technical anastomotic failure or severe disproportion between the target vessel and the graft, may lead to thrombosis, but vessel injury and hypercoagulability are among potential causes as well. 11

There was no evident inflammatory process in microscopy in our patient, but even if an inflammatory process was present preoperatively in our patient's SVs, the inflammatory background alone could not explain the dramatic intraoperative thrombosis. We hypothesized that increased thrombin generation and prothrombotic fibrin clot phenotype were responsible for the clinical presentation. Conversion of fibrinogen to fibrin (facilitated by thrombin) is a concluding step of coagulation. It has been shown that fibrin clots with small pores between tightly packed thin fibrin fibers are relatively lysis resistant. 12 Such clot phenotype has been evidenced in multiple thrombotic pathologies, such as myocardial infarction, 6 ischemic stroke, 13 and venous thromboembolism. 4 The prothrombotic clot phenotype, reflected by a tendency to form dense fibrin clots resistant to lysis, has been previously reported in patients with in-stent thrombosis. 14 While routine thrombophilia screening results in a high (almost 40%) detection rate of common hypercoagulable states, 15 there are prothrombotic conditions that escape routine diagnostic approach. The overall microscopic clot appearance and prothrombotic fibrin properties lead to the discovery of two mutations in our patient that are not routinely tested during thrombophilia screening, namely β-fibrinogen -455G > A and FXIII100G > T.

Elevated fibrinogen was postulated as one of the risk factors for early graft failure after CABG. 11 16 Epidemiological studies have established that elevated fibrinogen is strongly associated with cardiovascular diseases. 17 A 2005 meta-analysis of individual records of 154,211 participants from 31 prospective studies revealed that age- and sex-adjusted hazard ratio per 1 g/L increase in usual fibrinogen level for coronary heart disease was 2.4 (95% confidence interval [CI]: 2.2–2.6), while for stroke, the hazard ratio was as high as 2.1 (95%CI: 1.8–2.3). Risk of coronary disease progression was also linked to genetic polymorphisms of the fibrinogen gene. De Maat et al found that A allele of β-fibrinogen -455G > A was associated with more severe progression of coronary disease, as documented angiographically. 18 Gu and colleagues in a meta-analysis of 45 studies with 7,238 patients found that A allele of the β-fibrinogen -455G > A is associated with susceptibility to coronary disease, and, also with ischemic stroke (odds ratio for stroke = 1.5 [95% CI: 1.3–1.8] for AA + GA vs. GG). 19 In a recent study of patients with atrial fibrillation, Hu and colleagues found that the A allele of β-fibrinogen -455G > A was a risk factor for cardioembolic stroke, probably by elevating the level of plasma fibrinogen. 20 On the other hand, in a 2017 meta-analysis of 36 studies involving 26,940 cases and 34,694 controls, FXIII Val34Leu polymorphism was shown to be associated with risk myocardial infarction. 21 FXIII is crucial to thrombus stabilization, and changes of its plasma concentration reflect nonspecifically the extent of thrombosis, as shown by Li et al in a study on patients with cerebral venous thrombosis. 22 Interesting associations of FXIII Val34Leu polymorphism and thrombotic disorders have been reported. Jung et al reported in a meta-analysis of 11 studies that FXIII Val34Leu polymorphism is associated with recurrent pregnancy loss. 23 Although no association with the incidence of ischemic stroke was found for this polymorphism, 24 apparently when the stroke occurs, Val34Leu polymorphism of FXIII affects the severity of its outcome. 25 Furthermore, Kreutz and colleagues suggested in 2014 that FXIII Val34Leu polymorphism may increase risk of recurrent MI and death in patients with angiographically established coronary artery disease. 26 In 2009, our group has shown in a study of 113 patients that in patients undergoing CABG FXIII Leu34 allele is associated with decreased fibrin clot permeability and efficiency of fibrinolysis. 27

## Conclusion

Our extensive workup showed that β-fibrinogen HaeIII and FXIII Val34Leu polymorphisms are reflected in reduced clot permeability and susceptibility to lysis. These mutations likely contributed to intraoperative saphenous graft thrombosis. Further studies are needed to elucidate the role of these polymorphisms in early graft failure after bypass grafting procedures, however their contributory role seems evident.
